# Exocrine Pancreatic Insufficiency in Diabetes Mellitus: A Complication of Diabetic Neuropathy or a Different Type of Diabetes?

**DOI:** 10.1155/2011/761950

**Published:** 2011-08-01

**Authors:** Philip D. Hardt, Nils Ewald

**Affiliations:** Third Medical Department, University Hospital Giessen and Marburg, Giessen Site, Klinikstraße 33, 35392 Giessen, Germany

## Abstract

Pancreatic exocrine insufficiency is a frequently observed phenomenon in type 1 and type 2 diabetes mellitus. Alterations of exocrine pancreatic morphology can also be found frequently in diabetic patients. Several hypotheses try to explain these findings, including lack of insulin as a trophic factor for exocrine tissue, changes in secretion and/or action of other islet hormones, and autoimmunity against common endocrine and exocrine antigens. Another explanation might be that diabetes mellitus could also be a consequence of underlying pancreatic diseases (e.g., chronic pancreatitis). Another pathophysiological concept proposes the functional and morphological alterations as a consequence of diabetic neuropathy. This paper discusses the currently available studies on this subject and tries to provide an overview of the current concepts of exocrine pancreatic insufficiency in diabetes mellitus.

## 1. Introduction

Decades ago research groups interested in the pancreas demonstrated that pancreatic exocrine insufficiency (PEI) is present in a considerable percentage of patients with diabetes mellitus. These early studies were performed by direct pancreatic function tests (e.g., secretin-pancreozymin test). PEI was demonstrated not only in about 50% of patients with insulin-dependent diabetes (IDDM) but also in 30–50% of non-insulin-dependent diabetes (NIDDM) [1–6]. Apart from these functional studies it was also observed that pancreas morphology is altered in many patients with diabetes mellitus. In IDDM the weight of pancreata was reduced as compared to healthy controls [7, 8] and there were histological aspects of pancreatic atrophy [9]. Before the introduction of imaging techniques such as ultrasound, CT, and MRI, morphological studies of the pancreas in vivo were difficult. Therefore the collection of data was limited to very small numbers of patients. 

While in most of the discussions and comments the finding of altered pancreatic function and morphology was interpreted as a complication of diabetes mellitus, some scientists suggested that a high percentage of (previously not diagnosed) pancreatic diabetes might be a better explanation [2]. However, early studies on pancreatic function in diabetes were also limited to a rather small number of patients. This was due to the invasive nature of these test procedures including tube application into the duodenum and continuous aspiration of duodenal secretion for several hours.

In the last two decades of the 20th century indirect pancreatic function test became available. Especially the measurement of fecal elastase 1 concentrations (FECs) that can easily be performed in any setting proved rather good sensitivity and specificity [10–14]. Using these new indirect tests, the prevalence of PEI in diabetes mellitus could be demonstrated to be as high as in the earlier studies: nearly every second IDDM patient and one out of three NIDDM patient shows PEI [15–19]. The introduction of ultrasound, CT, and MRI did facilitate the investigation of pancreas morphology in vivo resulting in new research activity in the field. 

The features of modern diagnostic techniques of pancreatic function and morphology allowed the investigation of large patient cohorts. Therefore the reasons for PEI and morphological changes in diabetes can now be interpreted on a more solid data base.

In the present paper the relevant literature in this context is reviewed and discussed. If the thesis of a relevant percentage of pancreatic diabetes cases proves true, this will have major impact on our diagnostic and therapeutic strategies.

## 2. Material and Methods

A *Medline*-search was performed (March 14th, 2011) using the following search terms: (a) diabetes mellitus and exocrine pancreatic insufficiency (PEI); (b) diabetes mellitus and elastase 1; (c) diabetes mellitus and pancreatitis; (d) diabetes mellitus and exocrine pancreatic function; (e) pancreas morphology and diabetes. 

The search terms revealed the following number of quotations: (a) diabetes mellitus and exocrine pancreatic insufficiency (PEI): 279; (b) diabetes mellitus and elastase 1: 109; (c) diabetes mellitus and pancreatitis: 2321; (d) diabetes mellitus and exocrine pancreatic function, 1060; (e) pancreas morphology and diabetes: 9902.

Most of the papers addressed problems that are not directly related to the interest of the present review, for example diabetes and pancreatic cancer or pancreas/islet transplantation in diabetes mellitus. These were not further evaluated. The remaining articles were checked for the prevalence of PEI in diabetes mellitus, the prevalence of pancreatic diabetes (type 3c), morphological changes of the pancreas in diabetes mellitus, and the different hypotheses discussed in the context.

## 3. Results and Discussion

### 3.1. Pancreatic Exocrine Function in Diabetes Mellitus

Before the introduction of indirect function tests several studies have been performed using direct methods.  Table 1 shows the results reported in these. On average, PEI was reported in 52.4% (18–100%) of patients with diabetes mellitus. PEI was reported more often in patients with IDDM. However, in some of the studies it was observed equally often in NIDDM. In Table 2 the results of studies using indirect function tests are shown. On average 51% (26–74%) of patients with IDDM and 32% (28–36%) of patients with NIDDM showed abnormal exocrine function. While fecal elastase 1 concentrations (FECs) are considered a diagnostic tool for pancreatic function of unknown direct relevance for the clinical situation in terms of maldigestion and associated symptoms, it has also been shown in the meantime that in fact steatorrhea can be detected in 60% of patients with diabetes mellitus and reduced FEC [26]. In another previous study it was demonstrated that in 74% of patients classified as type 1 diabetes mellitus steatorrhea was present and steatorrhea correlated inversely with FEC concentrations [27]. While one follow-up study in only 20 patients claimed that a progression of PEI is rare in diabetes mellitus [28], it must be concluded from the information presently available that in patients with diabetes mellitus PEI is not only very frequent in terms of pathological function tests but also in terms of fat maldigestion. This finding might explain abdominal symptoms in patients with diabetes mellitus. Qualitative malnutrition (fat soluble vitamins) must also be considered. Most interestingly, the incretin action might be altered in patients with steatorrhea [29] as the secretion of incretins depends on the presence of digestive products inside the digestive tract [30].

### 3.2. Pancreatic Exocrine Morphology in Diabetes Mellitus

Autopsy studies and studies on pancreas histology showed marked changes in the exocrine gland in patients with diabetes mellitus as compared to nondiabetic controls. Blumenthal reported signs of chronic inflammatory changes of the exocrine pancreas in 11.2% of patients with diabetes mellitus as compared to 5.3% in nondiabetic patients [31]. In another autopsy study Olsen reported the percentage of exocrine pancreatic atrophy in diabetic patients to be 19% compared to 7% in controls [32]. In patients with long-lasting type 1 diabetes mellitus a reduced weight of the gland was reported [7, 8]. It was also observed that in IDDM patients there is histological evidence of exocrine pancreas atrophy [7, 9]. Even within only 24 hours after the diagnosis of type 1 diabetes mellitus, severe atrophy of exocrine tissue around beta cells without remaining insulin activity could be found while around beta cells with remaining insulin content no acinar atrophy could be observed [33]. Rahier et al. showed a clear reduction of weight in the glucagon-rich lobe, but no atrophy in the pancreatic-polypeptide-rich lobe despite the lack of beta cells (and therefore the lack of insulin) [34]. In clinical medicine changes of pancreatic duct morphology in patients with diabetes mellitus similar to the changes observed in chronic pancreatitis were observed at ERCP procedures [35, 36].

With the introduction of the abdominal ultrasound as a diagnostic tool, the investigation of the pancreas became easier. There are several studies reporting a reduced size of the pancreas in patients with diabetes mellitus. Compared to sex- and age-matched controls the pancreata of children and adolescents with type 1 diabetes mellitus appeared clearly smaller [37]. Similar ultrasound changes were demonstrated in adults with type 1 and type 2 diabetes mellitus [38] and by use of CT and MRI technology [39, 40].

### 3.3. Hypothesis Explaining Pancreatic Damage in Patients with Diabetes Mellitus

#### 3.3.1. Insulin as a Trophic Factor for Exocrine Tissue

In the second half of the last century it was shown that insulin acts as a trophic factor on acinar tissue [41]. There is a kind of portal vessel system supplying acinar cells directly with blood from nearby islets. Acinar cells located closely to these vessels are bigger and produce more enzymes than those located more distant [42]. But insulin is not only a local trophic factor, it also increases the enzyme output from cultivated islets [43]. In rats with glucose-stimulated insulin secretion the amount of total amylase and protein content is higher than that in diabetic rats [42]. From these observations in animal models and cell culture models it has been concluded that pancreatic atrophy as observed in some patients with diabetes mellitus might result from a lack of local trophic insulin effects. This hypothesis was supported by the fact that patients without any beta cell function display more obvious morphological changes than those with some residual insulin secretion [44]. Therefore it appears to be possible that local insulin depletion might explain morphological changes and exocrine dysfunction in some of the patients with diabetes mellitus. This concept was further supported by the observations of Cavalot et al. who reported a correlation between residual beta cell function and FEC [19]. However, if the local presence of insulin had such a major trophic effect, it is difficult to understand why about 50% of patients with type 1 diabetes do not show any relevant changes of the exocrine pancreas. Furthermore this hypothesis does not explain the findings in patients with type 2 diabetes mellitus since there is no local lack of insulin to be expected, at least in the early stages of the disease. 

In conclusion the concept of a lack of local trophic insulin action might explain some of the phenomena observed; however it cannot explain the whole phenomenon.

#### 3.3.2. Changes in Secretion/Action of Other Islet Hormones

Apart from the changes in insulin secretion or action, other islet cell hormones are also known to act differently in patients with diabetes mellitus as compared to healthy controls. Some of them play an important role in the regulation of digestive and metabolic functions and therefore they might also lead to a dysregulation of the exocrine pancreatic function [45]. As a consequence atrophy might result. A persisting elevation of glucagon levels as observed in some diabetic patients has been suggested to contribute to exocrine damage and dysfunction [46, 47]. Somatostatin is a relevant regulator of exocrine function [48], and elevated levels, as described in streptozotocin-induced diabetes mellitus, have been shown to reduce exocrine pancreatic function [49]. While changes in glucagon and somatostatin secretion might also contribute to exocrine damage in some of the patients with diabetes mellitus, they also do not fully explain the phenomena described in detail above. Some of the studies were only performed in animal models. and, more importantly, the patterns of secretory changes differ in type 1 and type 2 diabetes mellitus.

#### 3.3.3. Autoimmunity

Autoimmune pancreatitis has gained more attention previously. In the different types of the disease that have been described an involvement of both, exocrine and endocrine tissue, is very frequent. Diabetes mellitus associated with autoimmune pancreatitis can be treated by steroids, and it can even be cured if the autoimmune attack can be stopped [50, 51]. Apart from these cases exocrine antigens might also play a role in the induction of autoimmunity against beta cells in the absence of classical autoimmune diabetes. Some decades ago it has been demonstrated mainly from Japanese scientists that in some subtypes of diabetes mellitus antibodies are present which are directed against exocrine antigens. Kobayashi et al. described antibodies against pancreatic cytokeratin in 39% of recent onset patients classified as type 1 diabetes mellitus and in 20% of their relatives. In type 2 diabetes mellitus patients only 0.9% had relevant antibody titers. The authors suggested that the ultrastructure of exocrine tissue might be involved in the pathogenesis of type 1 diabetes mellitus [52]. Another antibody against exocrine tissue (BSDL) was found in 73.5% of patients at diagnosis of type 1 diabetes mellitus [53]. Imagawa et al. even suggested the introduction of a new diabetes type: patients with rapid loss of insulin secretion but without the presence of islet- or insulin-associated autoimmunity. On histological samples their patients displayed a marked inflammation in the exocrine tissue with elevated levels of pancreatic enzymes [54]. In another study antibodies against lactoferrin or carbonic anhydrase (both directed against exocrine targets) were present in 77% of patients classified as type 1 diabetes mellitus [55].

While the quantitative input of autoimmune pancreatitis associated with diabetes mellitus surely is rather small, the possible relevance of exocrine antigens in the pathogenesis of autoimmune diabetes deserves further attention.

#### 3.3.4. Diabetes Mellitus as a Consequence of Underlying Pancreatic Diseases

While PEI might be induced by diabetes mellitus, it could as well be the other way around as it has been proposed as early as 1963 by Chey et al. [2]. Different pancreatic diseases are known to induce diabetes mellitus, including different types of pancreatitis, pancreatic carcinoma, cystic fibrosis, and hemochromatosis. However, these diseases are believed to contribute to the burden of diabetes mellitus in only about 1% of all cases [56, 57]. The possibility that the prevalence of pancreatic diabetes might well be much higher than 1% has been discussed in detail elsewhere [58] and will not be issue of the present paper.

Additionally to those hypotheses explaining pancreatic damage in patients with diabetes mellitus, pancreatic exocrine insufficiency might also be interpreted as a complication of diabetic neuropathy.

### 3.4. Pancreatic Exocrine Insufficiency as a Complication of Diabetic Neuropathy

Diabetic neuropathy (DN) is a complication of diabetes mellitus that might occur in the time course of the disease. While the prevalence of DN is about 4–7.5% at the time of diagnosis, it is observed in 15–50% of the patients after 20–25 years of diabetes mellitus [59]. Autonomic neuropathy can affect not only the cardiovascular system, but also different functions of the digestive tract. Gastrointestinal motor disorders, for example, gastroparesis, diabetic diarrhea, diabetic obstipation, and fecal incontinence as a complication of diabetes mellitus have been described in detail [60]. It seems obvious that some of the gastrointestinal symptoms that occur in patients with diabetes mellitus are caused by DN. On the other hand, diabetic diarrhea and bloating might arise from the exocrine insufficiency that is probably even more common than DN in patients with diabetes mellitus. Since the regulation of enzyme synthesis and secretion does not only depend on gastrointestinal hormones produced somewhere else but also on local neurons and their signals, exocrine insufficiency itself could be caused by DN. This possibility has been taken into consideration in several studies dating back to the last century. However, one main argument against the assumption that DN might cause PEI was the lack of correlation between the duration of diabetes mellitus and the finding of exocrine insufficiency [2, 3]. However, more recently two studies [19, 61] demonstrated a significant increase of PEI in the time course of diabetes mellitus. Cavalot had shown earlier that fecal elastase 1 levels are correlated with the remaining C-peptide levels in 37 patients with type 1 diabetes mellitus suggesting that the remaining insulin presence might be of relevance for exocrine pancreatic function. While this explanation was in contrast to most other publications at that time, the finding could also be interpreted differently: C-peptide levels are known to decrease by time in type 1 diabetes mellitus while the probability of DN does increase. In fact, there was also a weak inverse correlation between diabetes duration and PEI despite the small number of individuals included in the study [26]. A more recent study by Ewald et al. involved data from 307 patients with different types of diabetes. Diabetes duration was inversely correlated with fecal elastase 1 concentrations (*P* = 0.004), and there was also a correlation between C-peptide levels and FEC (*P* < 0.001) [61]. The fact that earlier studies missed to show a correlation between disease duration and exocrine pancreatic function might be explained by smaller patient numbers that had been included into these studies.

Mechanisms linking exocrine pancreatic insufficiency might be the impairment of enteropancreatic reflexes or changes in gastrointestinal peptides. Enteropancreatic reflexes for example are known to play an important role in pancreatic responses, and according to older data as much as 50% of the exocrine pancreatic response to a meal may be mediated by enteropancreatic reflexes [22]. Interruption of the enteropancreatic reflexes by an autonomic neuropathy might therefore severely impair exocrine pancreatic function. Yet data on this topic is scarce and further research is strongly encouraged.

## 4. Conclusion

In the light of the literature reviewed for this paper there is clear evidence that both pancreas morphology and exocrine pancreatic function are very frequently and severely altered in patients with different types of diabetes mellitus. Several hypotheses have been discussed to explain these findings. A high prevalence of diabetes caused by pancreatic diseases (including autoimmune pancreatitis) appears unlikely because pancreatic diseases themselves have a low prevalence according to the clinical literature. Lack of local trophic insulin action causing acinar atrophy does not explain the fact that most patients with type 1 diabetes mellitus have no signs of exocrine changes. If local insulin deficiency was the main issue, an exocrine involvement should be expected in all patients with type 1 diabetes mellitus. The assumption that changes in hormonal secretion patterns in diabetic patients might explain exocrine pathology is also very unlikely for different reasons. However, as the prevalence of pancreatic exocrine insufficiency increases with decreasing C-peptide levels and over time, exocrine involvement might well be interpreted as a complication of diabetes mellitus that could be caused by diabetic neuropathy. At present, only very few studies directly interested in possible correlations between diabetic neuropathy and exocrine pancreatic dysfunction are available. There is one study by Vesterhus et al. describing a predominantly demyelinating neuropathy in a special diabetes type caused by a mutation in the CEL gene that includes pancreatic insufficiency. However, the presence of neuropathy was mainly attributed to the low vitamin D levels caused by PEI [62]. The second observation was made in a single case where neuropathy was also interpreted as a consequence of PEI in diabetes mellitus [63]. Larger studies on the association of diabetic neuropathy and exocrine pancreatic insufficiency should therefore be strongly encouraged.

## Figures and Tables

**Table 1:** Results of direct pancreatic function tests in patients with diabetes mellitus.

Author	Year	Subjects/diabetes type	Methods	Results
Pollard et al. [1]	1943	13	Amylase and lipase after pancreozymin-secretin stimulation	62% reduced
Chey et al. [2]	1963	50 diabetic patients; 13 juvenile type	Amylase and lipase after pancreozymin-secretin stimulation	Low amylase output in diabetes: 36%; in juvenile diabetes: 77%
Vacca et al. [3]	1964	55 diabetic patients (22 insulin treated)	Diastase and bicarbonate after secretin stimulation; fecal fat	73% abnormal; correlation with age, no correlation with fecal fat
Frier et al. [4]	1976	20 IDDM, 7 NIDDM, 13 controls	Stimulation with iv secretin and CCK-PZ	PEI: 80% IDDM; correlation with diabetes duration
Harano et al. [20]	1978	53 NIDDM, 4 IDDM, 18 controls	Secretin-pancreozymin test	Diabetes: 69% deficient enzyme output; correlation with diabetes control
Lankisch et al. [5]	1982	53 IDDM	Secretin-pancreozymin test	Diabetes: 43% impaired function
Bretzke et al. [21]	1984	60 insulin-treated type 2 diabetic patients	Secretin-pancreozymin test	Diabetes: 27% “mild PEI”
El Newihi et al. [22]	1988	10 type 2 diabetic patients with diarrhea and neuropathy	Secretin and CCK test	Enzyme and bicarbonate reduction in all subjects

**Table 2:** Results of indirect pancreatic function tests in patients with diabetes mellitus.

Author	Year	Subjects/diabetes type	Methods	Results
Hardt and Kloer [23]	1998	128 type 1+2	Fecal chymotrypsin Fecal elastase 1	45% <6 U/I 46% <200 *μ*g/g
Hardt et al. [15]	2000	39 type 1 77 type 2	Fecal elastase 1	74% <200 *μ*g/g 36% <200 *μ*g/g
Icks et al. [18]	2001	112 type 1	Fecal elastase 1	54.5% <200 *μ*g/g
Rathmann et al. [17]	2001	544 type 2	Fecal elastase 1	30.3% <200 *μ*g/g
Hardt et al. [16]	2003	323 type 1 697 type 2	Fecal elastase 1	51% <200 *μ*g/g 35% < 200 *μ*g/g
Nunes et al. [24]	2003	42 type 1+2	Fecal elastase 1	36% <200 *μ*g/g
Yilmaztepe et al. [25]	2005	32 type 2	Fecal elastase 1	28% <200 *μ*g/g
Cavalot et al. [19]	2004	66 type 1	Fecal elastase 1	26% <200 *μ*g/g
